# Mitochondrial NDUFA4L2 attenuates the apoptosis of nucleus pulposus cells induced by oxidative stress via the inhibition of mitophagy

**DOI:** 10.1038/s12276-019-0331-2

**Published:** 2019-11-18

**Authors:** Wen-Ning Xu, Huo-Liang Zheng, Run-Ze Yang, Tao Liu, Wei Yu, Xin-Feng Zheng, Bo Li, Sheng-Dan Jiang, Lei-Sheng Jiang

**Affiliations:** 1 Department of Clinic of Spine Center, Xinhua Hospital, Shanghai Jiaotong University School of Medicine, Shanghai, 200082 China; 2 Department of Orthopaedics, The Second Affiliated Hospital of Wenzhou Medical University, Wenzhou, Zhejiang Province China

**Keywords:** Apoptosis, Mitophagy

## Abstract

The main pathological mechanism of intervertebral disc degeneration (IVDD) is the programmed apoptosis of nucleus pulposus (NP) cells. Oxidative stress is a significant cause of IVDD. Whether mitophagy is induced by strong oxidative stress in IVDD remains to be determined. This study aimed to investigate the relationship between oxidative stress and mitophagy and to better understand the mechanism of IVDD in vivo and in vitro. To this end, we obtained primary NP cells from the human NP and subsequently exposed them to TBHP. We observed that oxidative stress induced mitophagy to cause apoptosis in NP cells, and we suppressed mitophagy and found that NP cells were protected against apoptosis. Interestingly, TBHP resulted in mitophagy through the inhibition of the HIF-1α/NDUFA4L2 pathway. Therefore, the upregulation of mitochondrial NDUFA4L2 restricted mitophagy induced by oxidative stress. Furthermore, the expression levels of HIF-1α and NDUFA4L2 were decreased in human IVDD. In conclusion, these results demonstrated that the upregulation of NDUFA4L2 ameliorated the apoptosis of NP cells by repressing excessive mitophagy, which ultimately alleviated IVDD. These findings show for the first time that NDUFA4L2 and mitophagy may be potential therapeutic targets for IVDD.

## Disc degeneration: Mitochondria at the root of the problem

A study in rats highlights the role of mitochondria in intervertebral disc degeneration (IVDD), one of the most important and prevalent predisposing factors for lower back pain. Previous studies have shown that in IVDD, oxidative stress results in the gradual loss of cells in the inner part of vertebral discs which cushion the space between vertebrae. Sheng-Dan Jiang and Lei-Sheng Jiang at Shanghai Jiaotong University School of Medicine found that oxidative stress in these cells causes the selective degradation of mitochondria by preventing the expression of a protein that is essential for mitochondrial function. Overexpressing this protein in the intervertebral discs of rats with IVDD alleviated degeneration, suggesting that restoring mitochondrial function could be an effective therapeutic strategy for easing the pain associated with the condition.

## Introduction

IVDD is one of the most common diseases that leads to a decline in quality of life and a high socioeconomic burden. IVDD is an important cause of low back pain, sciatica, and spinal stenosis. Approximately 80% of adult humans suffer from neck or back pain at some point in their lives^1^. However, the pathogenesis of IVDD has not been clearly illuminated.

Cellular redox status is involved in regulating metabolic signaling pathways in cells. This parameter is regulated by the rates of production and breakdown of reactive oxygen species (ROS)^2^. Reactive oxygen species (ROS) include superoxide anions, hydroxyl radicals and hydrogen peroxide^3^. ROS are second messengers in regulating metabolic signaling at the physiological level^4^. However, many ROS can damage proteins, DNAs and lipids, which induces autophagy and apoptosis^3^. Previous studies have reported that the overproduction of ROS destroys intervertebral disc (IVD) cells and disturbs the homeostasis of disc matrix^5,6^.

The programmed cell death (PCD) of IVD cells plays an important role in this process^7,8^. Apoptosis, a type of programmed cell death in IVDD, is a significant homeostatic mechanism in multicellular organisms, inducing the elimination of senescent cells and seriously damaged cells through an orderly process of cellular disintegration^9^. However, excessive apoptosis leads to a variety of diseases. Features of apoptosis include caspase activation, cell shrinkage, nuclear and cytoplasmic condensation, DNA fragmentation, and the formation of apoptosomes^10^. Apoptosis is thought to be a continuous process throughout life and plays a crucial role in IVDD progression.

Autophagy is a lysosomal degradation and self-digesting process. As one type of autophagy, mitophagy has an essential influence on the pathological and physiological functions of cells. Mitophagy is induced due to the damaged/dysfunctional mitochondria that safeguard cells against various cytotoxic stimuli via the removal of damaged/dysfunctional mitochondria^11^. However, excessive mitophagy eliminates many mitochondria, leading to apoptosis^12–14^. Our preliminary study indicated that oxidative stress activates mitophagy, whereas it is unclear how mitophagy is induced^15^.

Healthy intervertebral disc tissue is composed of an outer annulus fibrosus (AF) derived from the embryonic sclerotome and a central nucleus pulposus derived from the embryonic notochord, which survives in the physiologically hypoxic niche through the robust expression of hypoxia inducible factor (HIF)-1α. HIF-1α is essential for nucleus pulposus (NP) cell homeostasis and survival in a hypoxic environment. The conditional deletion of HIF-1α in NP cells causes cell death and the replacement of the NP by a biomechanically inferior fibrocartilaginous tissue^16^. Recent studies have shown that HIF-1α controls NDUFA4L2 expression by mediating mitochondrial function^17^. However, how the HIF-1α/NDUFA4L2 pathway regulates NP cells under oxidative stress conditions has not been explicitly clarified. We hypothesized that oxidative stress induces excessive mitophagy and that excessive mitophagy can cause apoptotic cell death in NP cells through the HIF-1α/NDUFA4L2 pathway.

## Materials and methods

### Cell isolation and culture

NP cells were obtained from human disc tissues and male Sprague–Dawley rats. Human disc tissues were obtained from patients who had undergone elective spinal surgery. Human nucleus pulposus tissue collection and experiments were approved by the Ethics Committee of Xinhua Hospital Affiliated with the Shanghai Jiao Tong University School of Medicine. All experiments involving human specimens followed the Helsinki declaration^18^. Rat disc tissues were obtained from male rats (6 weeks old and 200–250 g in weight). Briefly, fragments of human and rat disc tissues were treated with 0.1% collagenase for 4 h. After digestion, the tissues were partially removed as explants and placed in complete culture medium (DMEM/F12 and 10% fetal bovine serum (FBS) supplemented with antibiotics) at 37 °C in a 5% CO_2_ environment. NP cells moved out of the explants after 1 week. Finally, primary passage cells were harvested by 0.25% trypsin-EDTA (1 mM) solution and replanted in appropriate culture plates once confluent^19^. Human NP cells were used in follow-up experiments. Rat NP cells were used in the CHIP experiment.

### Cell viability assay

Cell viability was determined by the Cell Counting Kit-8 (CCK-8; C0037, Beyotime, China). NP cells were treated with TBHP at different concentrations (50, 100, 200, 400, and 800 μM) for 6 h or 400 μM for various times (0, 1, 3, 6, 12, and 24 h). Then, 100 μl of DMEM/F12 containing 10 μl of CCK-8 solution was added to each well after NP cells were washed with PBS. Subsequently, the cells were incubated for 1 h. The absorbance of the cells was detected by a microplate reader at 450 nm.

### siRNA and pc-NDUFA4L2 plasmid transfection

Small interfering RNAs specifically targeting HIF-1α and NDUFA4L2 were designed and synthesized chemically (Gene Pharma, China). The sequences of si-HIF-1α-1 and si-HIF-1α-2 were 5′-UUCUCCGAACGUGUCACGUTT-3′ (sense) and 5′-ACGUGACACGUUCGGAGAATT-3′ (antisense) and 5′-GGGCCGUUCAAUUUAUGAATT-3′ (sense) and 5′-GCCUCUUCGACAAVCUUAATT-3′ (antisense), respectively. The sequences of si-NC and si-NDUFA4L2 were 5′-UUCUCCGAACGUGUCACGUTT-3′ (sense) and 5′-ACGUGACACGUUCGGAGAATT-3′ (antisense) and 5′-CCCGCUUCUACCGGCAGAUTT-3′ and 5′-GGAACCGCAUGAGUCCCAATT-3′, respectively. si-HIF-1α and si-NDUFA4L2 were transfected into NP cells. In brief, si-HIF-1α was added to serum-free medium, and then, Lipofectamine 2000 (Invitrogen, Carlsbad, California, CA) was added to the same serum-free medium. The mixtures were incubated for 20 min. Finally, the mixtures were added to plates. pcDNA-NDUFA4L2 plasmids were designed and synthesized chemically (Sangon Biotech, China). The cloning vector was pcDNA3.1 + . As described above, NDUFA4L2 plasmids were added to serum-free medium, and then, Lipofectamine 2000 (Invitrogen, Carlsbad, CA) was added to the same medium. All procedures were performed using Lipofectamine 2000 according to the manufacturer’s instructions.

### Detection of apoptosis by flow cytometry

Apoptosis was detected by using an Annexin V-FITC apoptosis detection kit (Beyotime). Cell samples were obtained from 60-mm culture plates after treatment with TBHP. Then, they were transferred to 15-ml centrifuge tubes and resuspended in 195 µl of 1× binding buffer. Next, 5 µl of Annexin V-FITC and 10 µl of PI were added. The samples were gently vortexed and incubated for 20–30 min at room temperature in the dark. The samples were immediately analyzed by flow cytometry.

### Western blot analysis

NP cells were seeded on 60-mm culture plates at a density of 6 × 10^5^ cells/well. The cells were treated with TBHP (400 μM). These concentrations were chosen based on our experiments and our previous studies. After exposure to various treatments, NP cells were lysed in RIPA buffer (Beyotime, Haimen, China) at 4 °C for 0.5 h. The cell lysates were stored at −80 °C. The total protein concentration was detected by the Bradford method. The samples were then separated by SDS-polyacrylamide gel electrophoresis and electroblotted onto polyvinylidene difluoride membranes. The membranes were blocked with TBST-buffered saline solution containing 5% dry milk for 2 h and then incubated overnight at 4 °C or at 25 °C for 2 h with primary antibodies against HIF-1α (1:1000, Proteintech, China), Bcl-2 (1:1000, Proteintech, China), Bax (1:1000, Proteintech, China), Beclin-1 (1:1000, CST, USA), LC-3 (1:1000, Novus, USA), cleaved caspase-3 (1:1000, Proteintech, China), cleaved PARP (1:1000, CST, USA), NDUFA4L2 (1:1000, Proteintech, China), and P62 (1:1000, Proteintech, China). Thereafter, the membranes were incubated with corresponding horseradish peroxidase-conjugated secondary antibodies at 25 °C for 1 h. The membranes were detected with ECL plus reagent (Millipore) using the ChemiDoc^TM^ XRS + System (Bio-Rad, USA). Relative proteins of interest were analyzed based on pixel density with Gel-Pro analyzer software (Media Cybernetics, Inc., Rockville, MD, USA) and then normalized to a corresponding GAPDH loading control prior to statistical analyses.

### Measurement of the mitochondrial membrane potential

The mitochondrial membrane potential (mtΔΨ) was detected by a JC-1 kit (C2006; Beyotime, China) according to the manufacturer’s instructions. Briefly, after treatment with TBHP for the time indicated, the NP cells were obtained and resuspended in 500 µl of JC-1 staining soluted and then incubated in the dark at 37 °C for 20 min. The cells were resuspended in 500 µl of ice-cold staining buffer and detected by flow cytometry after two washes with ice-cold staining buffer and centrifugation. In normal cells, mitochondria had a high mtΔΨ, and JC-1 formed orange-red fluorescent J-aggregates, while in cells with depolarized or damaged mitochondria, the sensor dye appeared as green fluorescent monomers. The value of the mtΔΨ from each sample is represented as the ratio of red fluorescence intensity to green fluorescence intensity.

### Detection of apoptotic cells by Hoechst 33258 staining

Apoptotic NP cells were identified by Hoechst 33258 staining (Beyotime, Haimen, China). NP cells were seeded at a density of 6 × 10^5^ cells/well in a 60-mm plate. After treatment with TBHP and other drugs, NP cells were fixed with 4% paraformaldehyde for 15 min, washed with PBS three times and stained with 2 µg/ml Hoechst 33258 (Beyotime, Haimen, China) in Hank’s balanced salt solution for 5 min. The morphologic changes in apoptotic nuclei were assessed under a fluorescence microscope (Olympus Fluoview, Tokyo, Japan) with excitation at 350 nm and emission at 460 nm.

### MRFP-GFP-LC-3 adenovirus transfection

Approximately 1 × 10^5^ NP cells per well were seeded on sheet glasses in 24-well and cultured at 37 °C until cells reached a concentration of 2 × 10^5^ cells/well. After polybrene was added to the medium, 1 μM mRFP-GFP-LC-3 adenovirus was transfected into NP cells and incubated for 48 h. NP cells transfected with mRFP-GFP-LC-3 adenovirus were used for follow-up experiments. The green dot represents the initial autophagosome, and the red dot represents the mature autophagosome.

### Coimmunofluorescence analysis

Immunofluorescence analysis was performed according to a previous study^20^. NP cells were plated on sheet glasses in six-well plates and then used for follow-up experiments. For LC-3, NDUFA4L2 and cytochrome C, NP cells were fixed in 4% paraformaldehyde for 20 min and permeabilized with 0.1% Triton X-100 for 15 min. The cells were blocked in 5% bovine serum albumin in PBS for 1 h. The sheet glasses were incubated with primary antibodies overnight at 4 °C. Primary antibodies against LC3 and NDUFA4L2 were incubated together, and cytochrome c and NDUFA4L2 antibodies were incubated together. The primary antibodies were diluted as follows: LC3 mouse polyclonal antibody was diluted 1:100 (Santa Cruz, USA), NDUF4L2 rabbit polyclonal antibody was diluted 1:100 (Proteintech, China) and cytochrome c mouse polyclonal antibody was diluted 1:100 (Proteintech, China). The sheet glasses containing NP cells were washed with PBST three times. Then, the NP cells were incubated with Cy3-conjugated AffiniPure goat anti-rabbit IgG (H + L) and Alexa Fluor 488-conjugated AffiniPure goat anti-mouse IgG (H + L). Immunofluorescence was observed by fluorescence microscopy (Olympus BX51).

### Chromatin immunoprecipitation (CHIP) assays

NP cells were transfected with si-NDUFA4L2 or si-NC before being exposed to TBHP. CHIP was carried out according to a previous study^21^. NP cells were fixed with 1% formaldehyde to crosslink chromatin protein (HIF-1α) to DNA for 10 min. The specimens were lysed by ultrasonication and incubated with specific antibodies overnight at 4 °C. DNA eluted by HIF-1α was analyzed by PCR. The results of CHIP were analyzed by the fold enrichment method, and at least three independent experiments were performed.

### Transmission electron microscopy

NP cells treated with TBHP were fixed in 2.5% glutaraldehyde overnight. Thereafter, we used 2% uranyl acetate to stain NP cells for 1 h followed by postfixation in 2% osmium tetroxide for 1 h. After scratching and embedding in Durcopan ACM for 6 h, these samples were stained with uranyl acetate and lead citrate and then assessed with a Zeiss EM900 transmission electron microscope (Gottingen, Germany).

### Surgical procedure

Rat experiments were conducted according to International Guiding Principles for Biomedical Research Involving Animals and were approved by our University Ethics Committee (Ethics Committee of Xinhua Hospital Affiliated with Shanghai Jiao Tong University School of Medicine). Fifteen one-year-old healthy male adult rats were randomly divided into three groups: the sham operation group (skin incision and injection of control adenovirus, sham + NC group), the unbalanced dynamic group (surgical procedure and injection of control adenovirus, IVDD + NC group), and the treatment group (unbalanced dynamics and injection of NDUFA4L2 adenovirus, IVDD + NDUFA4L2 group). To establish a model of lumbar IVDD, the unbalanced dynamic group and treatment group underwent a surgical procedure after anesthesia, as described previously^22^. Briefly, the rats were anesthetized with 0.1 ml of 1% pentobarbital (50 mg/kg) administered intraperitoneally. After 5 min, the spinous processes, sacrospinal muscles, interspinous ligaments, supraspinous ligaments, and posterolateral 1/2 of bilateral zygapophysial joints of the lumbar spine were removed through a dorsal medial approach. The sham operation group underwent skin incision as a control. All rats were housed in the same environment (22 °C, sufficient food and water and a 12-h light and 12-h dark cycle). Six months after surgery, the disc tissues were collected.

### NDUFA4L2 adenovirus transfection in vivo

NDUFA4L2 adenovirus transfection was described previously^1^. After the surgical procedure was performed, the L4/L5 discs were confirmed by a trial radiograph. The L4/L5 discs were punctured through the annulus fibrosus by needles. To avoid deep punctures, we adapted the length of needles based on the dimensions of the nucleus pulposus and the annulus fibrosus, which were defined as ~4 mm by a preliminary experiment. The needles were fixed into the nucleus pulposus, and adenovirus was then injected into the nucleus pulposus every month until the rats were sacrificed. Finally, the needles were pulled out gently and slowly.

### Immunohistochemical examination

Six months after surgery, the disc tissues were embedded in paraffin. The human and rat specimens were fixed in formaldehyde, dehydrated in gradient solutions of ethyl alcohol and embedded in paraffin. Subsequently, the tissues were cut into 5-μm sections continuously. These sections were deparaffinized and rehydrated. The rehydrated sections were microwaved in 0.01 mol/L sodium citrate for 15 min each. Next, endogenous peroxidase activity was blocked by 3% hydrogen peroxide for 10 min, and nonspecific binding sites were blocked by 5% bovine serum albumin for 30 min at RT. The sections were then incubated with antibodies against HIF-1α (anti-HIF-1α, 1:200), Parkin (anti-Parkin, 1:200), cleaved caspase-3 (anti-cleaved caspase-3, 1:200), and NDUFA4L2 (anti-NDUFA4L2, 1:100) overnight at 4 °C. Finally, the sections were incubated in an HRP-conjugated secondary antibody (Santa Cruz Biotechnology, Dallas, TX, USA). After counterstaining with hematoxylin, the sections were observed under a microscope, and images were obtained via Image-Pro Plus software version 6.0 (Media Cybernetics, Rockville, MD, USA). Each specimen was cut into at least three sections to analyze the expression of HIF-1α and NDUFA4L2.

### Safranin O-fast green

Six months after surgery, the disc tissues were embedded in paraffin. The disc specimens from the rats were decalcified in EDTA and postfixed in formaldehyde. After being fixed in formaldehyde, the disc specimens from the rats were dehydrated in gradient solutions of ethyl alcohol and then embedded in paraffin. The specimens were cut into 5-μm sections. Each rat disc was stained with safranin O-fast green (S-O). Experienced histological researchers evaluated the morphology of the discs in a blinded manner.

### Magnetic resonance imaging method

The rat lumbar spine was assessed by magnetic resonance imaging (MRI) 6 months after the surgical procedure. Changes in the signal and structure of sagittal T2-weighted images were assessed by a 3.0 T clinical magnet (Philips Intera Achieva 3.0MR). The sagittal T2-weighted sections were obtained with the following settings: fast-spin echo sequence with time to repetition (TR) of 5400 ms and time to echo (TE) of 920 ms; 320 (h) 9 256 (v) matrix; field of view of 260; and 4 excitations. The section thickness was 2 mm with a 0-mm gap. The MRIs were evaluated by another blinded orthopedic researcher using the classification of intervertebral disk degeneration as reported by Pfirrmann et al. 43 (1 point = grade I, 2 points = grade II, 3 points = grade III, 4 points = grade IV).

### Statistical analysis

The data are shown as the mean ± SD (standard deviation) of at least three independent experiments. Statistical analyses were performed with the SPSS 18 statistical software program (SPSS, Inc., Chicago, IL). Multiple comparisons of data among the groups were carried out by one-way ANOVA and Tukey’s post hoc test. Independent-samples *t*-tests were used to analyze the differences between two groups. Differences were considered statistically significant when the *P* value was <0.05. ***p < 0.001, **p < 0.01, *p < 0.05.

## Results

### HIF-1α is involved in autophagy and apoptosis induced by oxidative stress in the nucleus pulposus

**Fig. 1:**
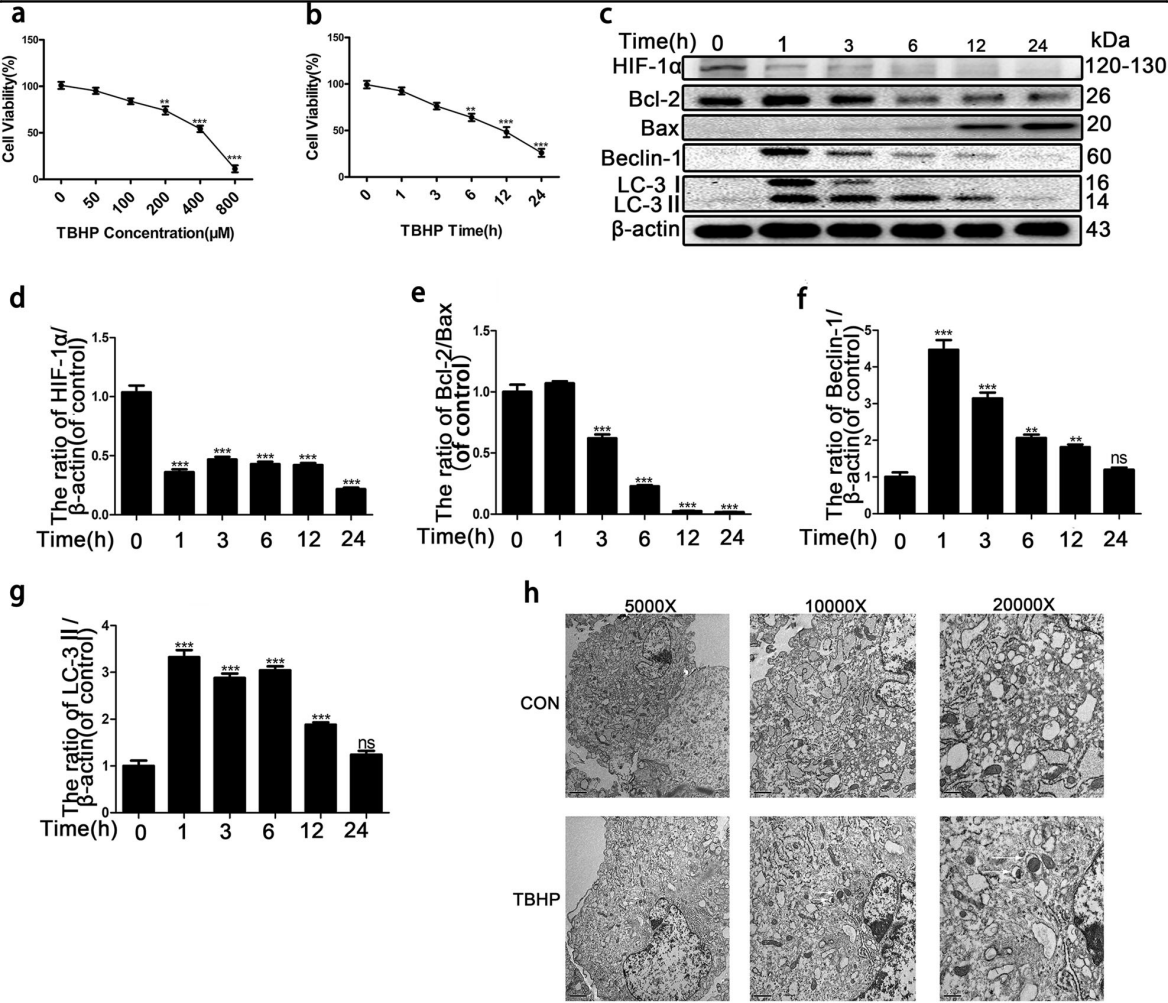
HIF-1α is involved in autophagy and apoptosis induced by oxidative stress in the nucleus pulposus. Primary nucleus pulposus cells were cultured in 400 μM TBHP for a prolonged time. **a**, **b** Cell viability was determined by the CCK-8 assay. **c** Western blotting for the protein levels of HIF-1α, Bcl-2/Bax, Beclin-1, and LC-3II. **d–g** Quantitative analysis of the protein contents of HIF-1α, Bcl-2/Bax, Beclin-1 and LC-3II. **h** Transmission electron microscopy was used to identify autophagosomes and autophagolysosomes. The data are represented as the mean ± S.D. ***p < 0.001, **p < 0.01, *p < 0.05 (*n* = 5).

NP cell apoptotic death induced by oxidative stress is a main factor of IVDD^23,24^. First, we confirmed the cytotoxicity of TBHP in NP cells at different concentrations (50, 100, 200, 400, and 800 μM) for 6 h. The CCK8 assay showed that cell viability was reduced to 95.2%, 84.0%, 74.0%, 54.3%, and 11.3%, respectively (Fig. 1a). Flow cytometry analysis of Annexin V-FITC/PI staining and Hoechst 33258 staining revealed that the percentage of apoptotic NP cells with surface-associated annexin-V staining (early apoptosis plus late apoptosis) and nuclear condensation gradually increased with increasing concentrations of TBHP treatment (50, 100, 200, 400, and 800 μM) for 6 h (Fig. S1a-c). Western blotting also showed that the ratio of Bcl-2/Bax was decreased (Fig. S1d and f). Second, the NP cells were treated with 400 μM TBHP for various times (0, 1, 3, 6, 12, and 24 h). The CCK8 assay showed that cell viability was reduced to 92.6%, 76.2%, 64.1%, 48.3%, and 26%, respectively (Fig. 1b). The ratio of Bcl-2/Bax decreased as the time increased (Fig. 1c, e). These results confirmed that oxidative stress induced by TBHP caused the apoptotic death of NP cells.

Excessive autophagy is involved in apoptosis^12^. Our previous study also affirmed that excessive autophagy causes the apoptosis of NP cells^23^. To determine the level of autophagy in NP cells cultured with TBHP, we detected the protein levels of LC3-II and Beclin-1, which are indicators of autophagy formation, by Western blotting. The protein level of LC3-II, as well as the protein expression of Beclin-1, was increased 6 h after treatment with TBHP in a dose-dependent manner (Fig. S1d, g and h). Subsequently, we detected the protein levels of LC3-II and Beclin-1 after prolonged TBHP treatment (400 μM). The protein levels of LC3-II and Beclin-1 also increased to the highest point at 3 h and 6 h (Fig. 1c, f, g). Therefore, these results indicated that there was a relationship between autophagy and apoptosis. To further confirm that autophagy was increased in NP cells exposed to 400 μM TBHP for 6 h, transmission electron microscopy was used to detect autophagosomes, and the results showed that autophagosomes were observed in NP cells exposed to 400 μM TBHP for 6 h (Fig. 1h). Ultimately, 400 μM TBHP for 6 h was used in all subsequent experiments.

Some studies have reported that HIF-1α regulates the apoptosis of cells^17^. Additionally, our previous studies reported that HIF-1α, expressed largely in intervertebral cells, plays a significant role in the metabolism and survival of intervertebral disc cells. Our results also confirmed that the HIF-1α protein is expressed largely in NP cells^25^. Here, the expression of HIF-1α was decreased in NP cells (Fig. 1c, d; Fig. S1d and e). However, the role of HIF-1α in the apoptosis of NP cells caused by oxidative stress remains unknown.

### Excessive mitophagy causes apoptotic cell death in NP cells after treatment with TBHP

**Fig. 2:**
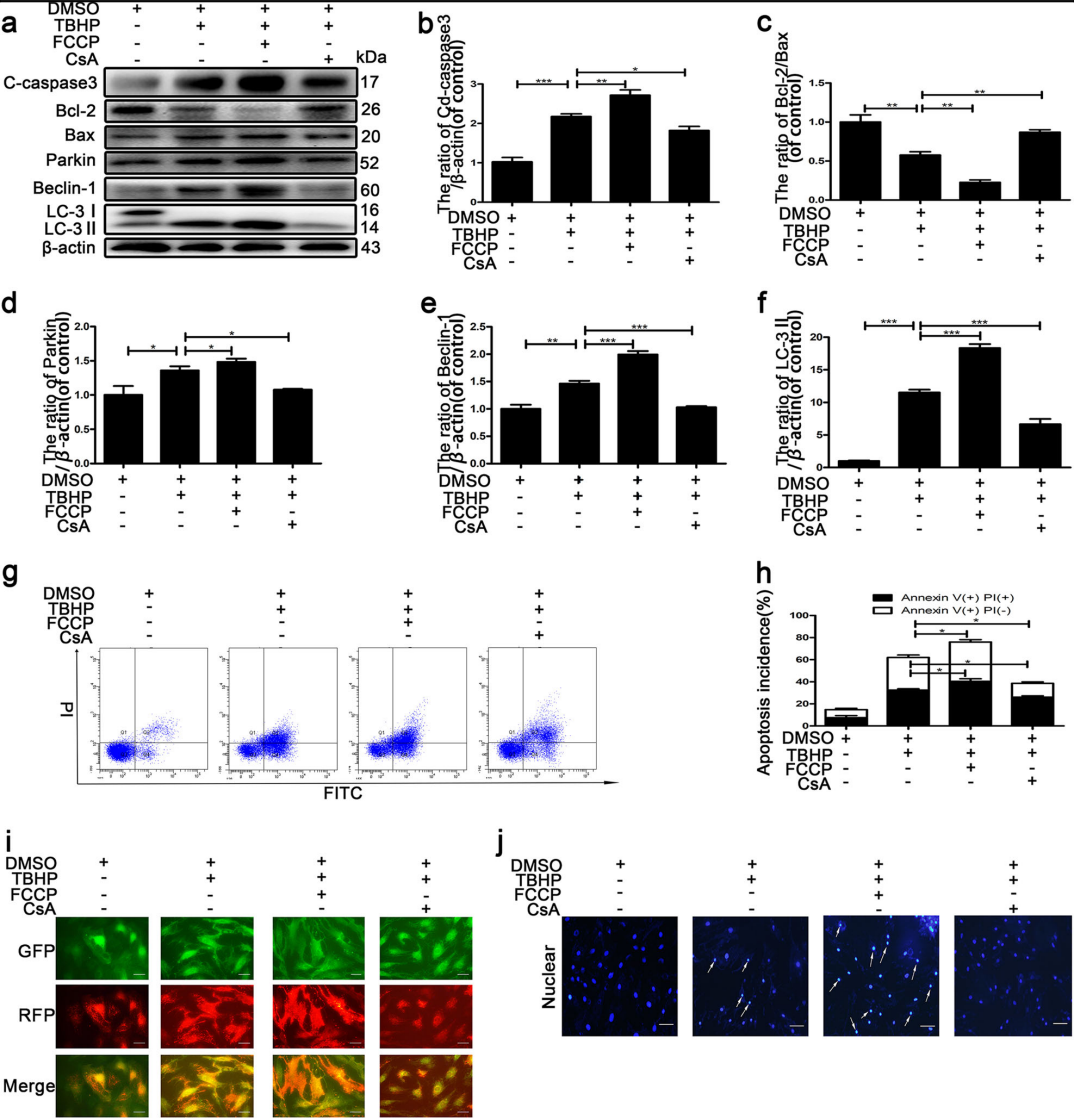
Excessive mitophagy accelerates NP cell apoptosis. After pretreatment with FCCP or CsA, primary nucleus pulposus cells were exposed to 400 μM TBHP for 6 h. **a** Western blotting for the protein levels of cleaved caspase-3, Bcl-2/Bax, Beclin-1, Parkin and LC-3II. **b–f** Quantitative analysis of the protein content of cleaved caspase-3, Bcl-2/Bax, Beclin-1, Parkin and LC-3II. **g** Flow cytometry was used to detect apoptosis in nucleus pulposus cells. **h** Quantitative analysis of flow cytometry for the detection of apoptosis. **i** Fluorescence images of NP cells infected with mRFP-GFP-LC-3 adenovirus. sensGFP is sensitive to the pH changes owing to the fusion of autophagosomes and lysosomes, whereas mRFP is stable. When autophagy was induced, autophagosomes and lysosomes were fused, sensGFP was quenched and mRFP was increased. **j** Hoechst 33258 staining detected apoptosis in the cell nucleus. Nuclear condensation was observed in apoptotic cells. The data are presented as the mean ± S.D. ***p < 0.001, **p < 0.01, *p < 0.05 (*n* = 5). Cd caspase-3: cleaved caspase-3.

Our previous study suggested that excessive autophagy causes the apoptosis of NP cells^23^. We hypothesized that autophagy causes the apoptosis of NP cells through mitophagy. To confirm whether mitophagy causes apoptotic cell death in NP cells, we treated NP cells with the mitophagy promoter FCCP (carbonyl cyanide 4-(trifluoromethoxy) phenylhydrazone) (1 μM)^26^ and the inhibitor CsA (cyclosporin A) (5 μM)^27^ 1 h prior to exposing the NP cells to TBHP. As shown in Fig. 2a–f, we observed that the protein levels of LC3-II, the mitophagy marker Parkin and Beclin-1 were increased under FCCP pretreatment. However, pretreatment with CsA significantly decreased the protein levels of LC3-II, Beclin-1 and the mitophagy marker Parkin. Immunofluorescence also showed that mitophagy was repressed by CsA and facilitated by FCCP (Fig. 2i). Then, we evaluated the ratio of apoptotic cell death in NP cells by flow cytometry analysis, Western blotting and Hoechst 33258 staining (Fig. 2a, g, h, j). Flow cytometry analysis showed that the ratio of apoptotic cell death was higher upon pretreatment with FCCP, while it was lower upon pretreatment with CsA compared with TBHP treatment (Fig. 2g, h). Western blotting revealed that FCCP markedly increased the expression of an apoptosis-related protein (cleaved caspase-3) and the Bcl-2/Bax ratio, but CsA attenuated TBHP cytotoxicity (Fig. 2a–c). Meanwhile, the evaluation of morphology by Hoechst 33258 staining showed that there were more apoptotic cells with nuclear condensation than those found with TBHP treatment only (Fig. 2j). Additionally, decreased apoptotic cell death with nuclear condensation was observed in NP cells pretreated with CsA. These results suggested that TBHP induced apoptotic cell death via mitophagy in NP cells.

### HIF-1α plays a key role in TBHP-induced apoptotic cell death in NP cells

**Fig. 3:**
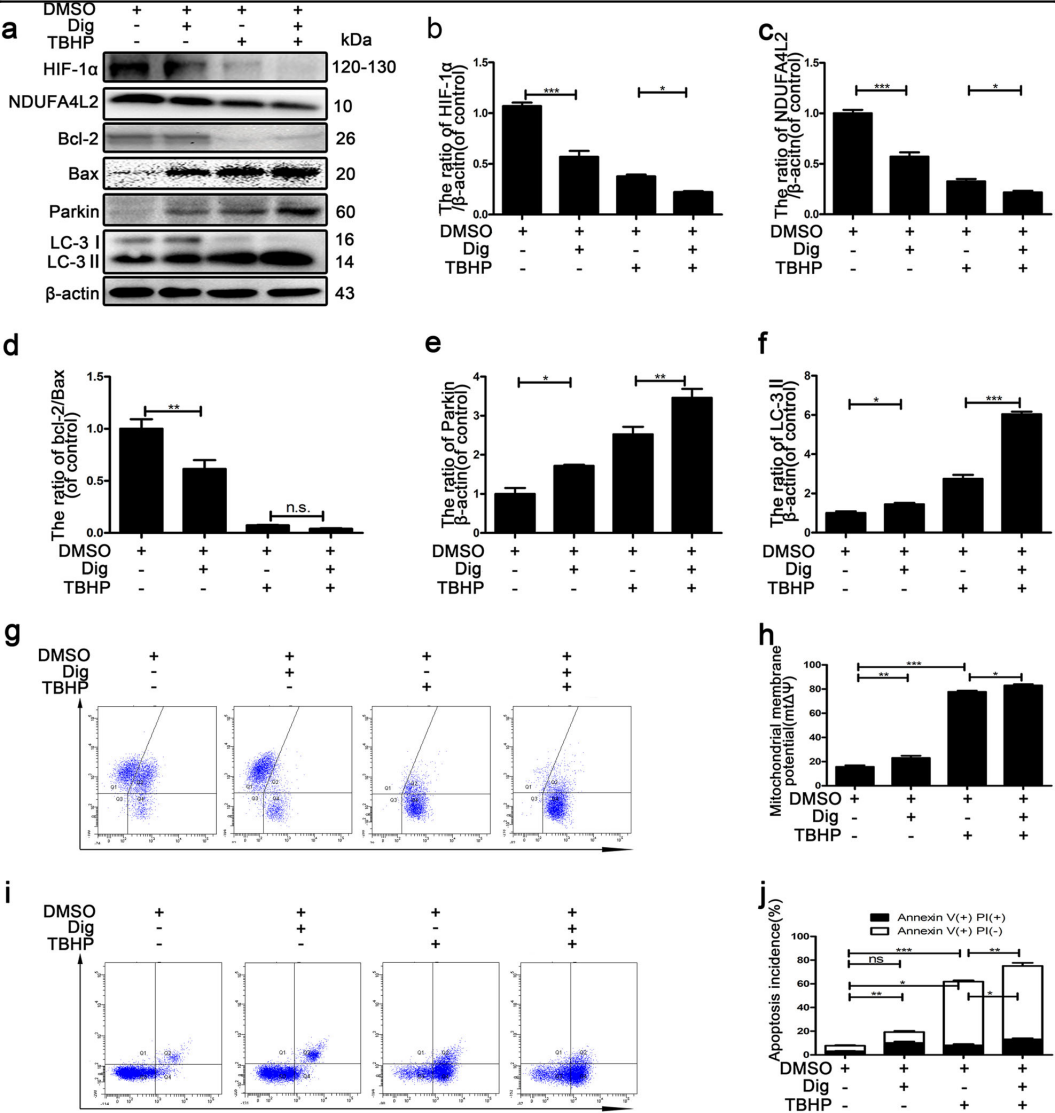
HIF-1α plays an important role in apoptosis induced by mitophagy via NDUFA4L2. Primary nucleus pulposus cells were pretreated with Dig before being exposed to TBHP. **a** Western blotting for the protein levels of HIF-1α, NDUFA4L2, Bcl-2/Bax, Parkin and LC-3II. **b–f** Quantitative analysis of the protein contents of HIF-1α, NDUFA4L2, Bcl-2/Bax, Parkin and LC-3II. **g** Flow cytometry was used to determine the mitochondrial membrane potential (mtΔΨ) by JC-1 staining. **h** Quantitative analysis of the mitochondrial membrane potential (mtΔΨ). (**i**) Flow cytometry was used to detect apoptosis in nucleus pulposus cells. **j** Quantitative analysis of flow cytometry for the detection of apoptosis. The data are presented as the mean ± S.D. ***p < 0.001, **p < 0.01, *p < 0.05 (*n* = 5).

Another study suggested that hypoxia protects NP cells against apoptosis caused by serum-free medium via restraining excessive autophagy. Here, to investigate whether the inhibition of HIF-1α causes excessive autophagy flux to induce apoptotic cell death in NP cells, we used the HIF-1α inhibitor digoxin (100 nM)^28^ to inhibit HIF-1α. Digoxin was added to the medium for 1 h before TBHP treatment. As shown in Fig. 3a, e, f, NP cells exhibited further accumulation of LC-3II and Parkin. We also found that the protein expression of LC3-II and Parkin was increased after treatment with digoxin alone in NP cells. The ratio of Bcl-2/Bax was not increased by digoxin pretreatment (Fig. 3a, d), but flow cytometry analysis demonstrated that digoxin promoted the apoptosis of NP cells (Fig. 3i, j). Importantly, the protein expression of NDUFA4L2 and HIF-1α was decreased by digoxin pretreatment (Fig. 3a–c). The results of JC-1 staining showed that the mitochondrial membrane potential was reduced in NP cells exposed to TBHP, and Dig further reduced the mitochondrial membrane potential (Fig. 3g, h). Recent studies have reported that mitochondrial NDUFA4L2 regulates the apoptosis of cardiomyocytes^29^. These findings suggested that HIF-1α regulated the metabolism and survival of NP cells through mitochondrial NDUFA4L2. Mitochondrial NDUFA4L2 regulated oxidative phosphorylation by ECL, which had a beneficial influence on the mitochondrial membrane potential. Importantly, an immunofluorescence assay showed that NDUFA4L2 was located in mitochondria in NP cells (Fig. S2a).

**Fig. 4:**
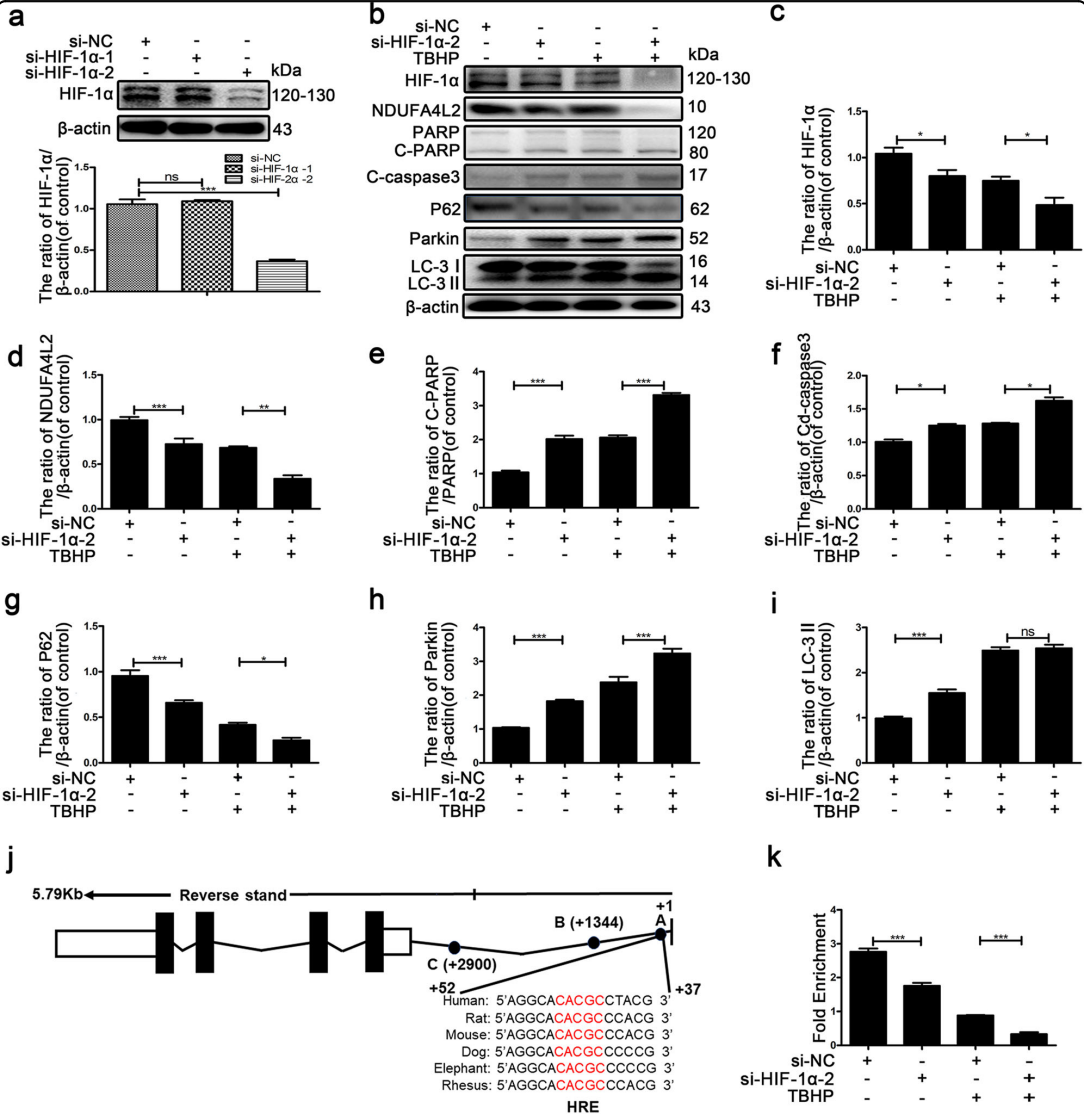
Silencing of HIF-1α promoted the apoptosis of NP cells through NDUFA4L2. Primary nucleus pulposus cells were transfected with si-HIF-1α or si-NC for 48 h and then used for follow-up experiments. **a** Western blotting of the protein level of HIF-1α was performed to determine the effect of knockdown. **b** Western blotting for the protein levels of HIF-1α, NDUFA4L2, cleaved PARP/total PARP, cleaved caspase-3, P62, Bcl-2/Bax, Parkin and LC-3II. **c–i** Quantitative analysis of the protein contents of HIF-1α, NDUFA4L2, cleaved PARP/total PARP, cleaved caspase-3, P62, Bcl-2/Bax, Parkin and LC-3II. **j** A schematic representation of the NDUFA4L2 gene and the nucleotide sequences matching consensus hypoxia response elements (HREs) from six mammalian genes, indicating the regions analyzed in the CHIP assay. **k** CHIP assay for HIF-1α binding to the NDUFA4L2 gene in NP cells. The data are presented as the mean ± S.D. ***p < 0.001, **p < 0.01, *p < 0.05 (*n* = 5). Cd PARP: cleaved PARP.

Next, we knocked down HIF-1α by si-HIF-1α. The results in Fig. 4a show that HIF-1α was knocked down by si-HIF-1α-2. The silencing of HIF-1α obviously increased the protein expression of LC3-II and Parkin and decreased the protein expression of P62 (Fig. 4b, c, g, h and i). These findings indicated that the inhibition of HIF-1α resulted in excessive mitophagy. Finally, we detected the level of apoptotic NP cell death by Western blotting. Compared with si-HIF-1α alone, the Bcl-2/BAX ratio was lower. Cleaved PARP and cleaved caspase-3 expression was promoted in NP cells transfected with si-HIF-1α-2 (Fig. 4b, e, and f). Notably, the protein expression of mitochondrial NDUFA4L2 was decreased in NP cells containing si-HIF-1α-2 (Fig. 4b, d). To further confirm possible hypoxia response elements (HREs) of NDUFA4L2, the proximal promoter region (intron 1) of NDUFA4L2 was predicted by nucleotide sequence matching. The highly conserved sequence is at the + 43 site between different species. CHIP (chromatin immunoprecipitation) assays were performed on human NP cells (Fig. 4j) and rat NP cells (Fig. S3a). To detect the level of HIF-1α binding to NDUFA4L2’ HREs, qPCR was carried out with specific primers for the HRE site (A). The level of HIF-1α binding to NDUFA4L2′ HREs was decreased in NP cells exposed to TBHP (Fig. 4k). These results revealed that mitochondrial NDUFA4L2 was involved directly in HIF-1α-regulated oxidative stress.

### Inhibition of mitochondrial NDUFA4L2 by oxidative stress causes excessive mitophagy in NP cells

**Fig. 5:**
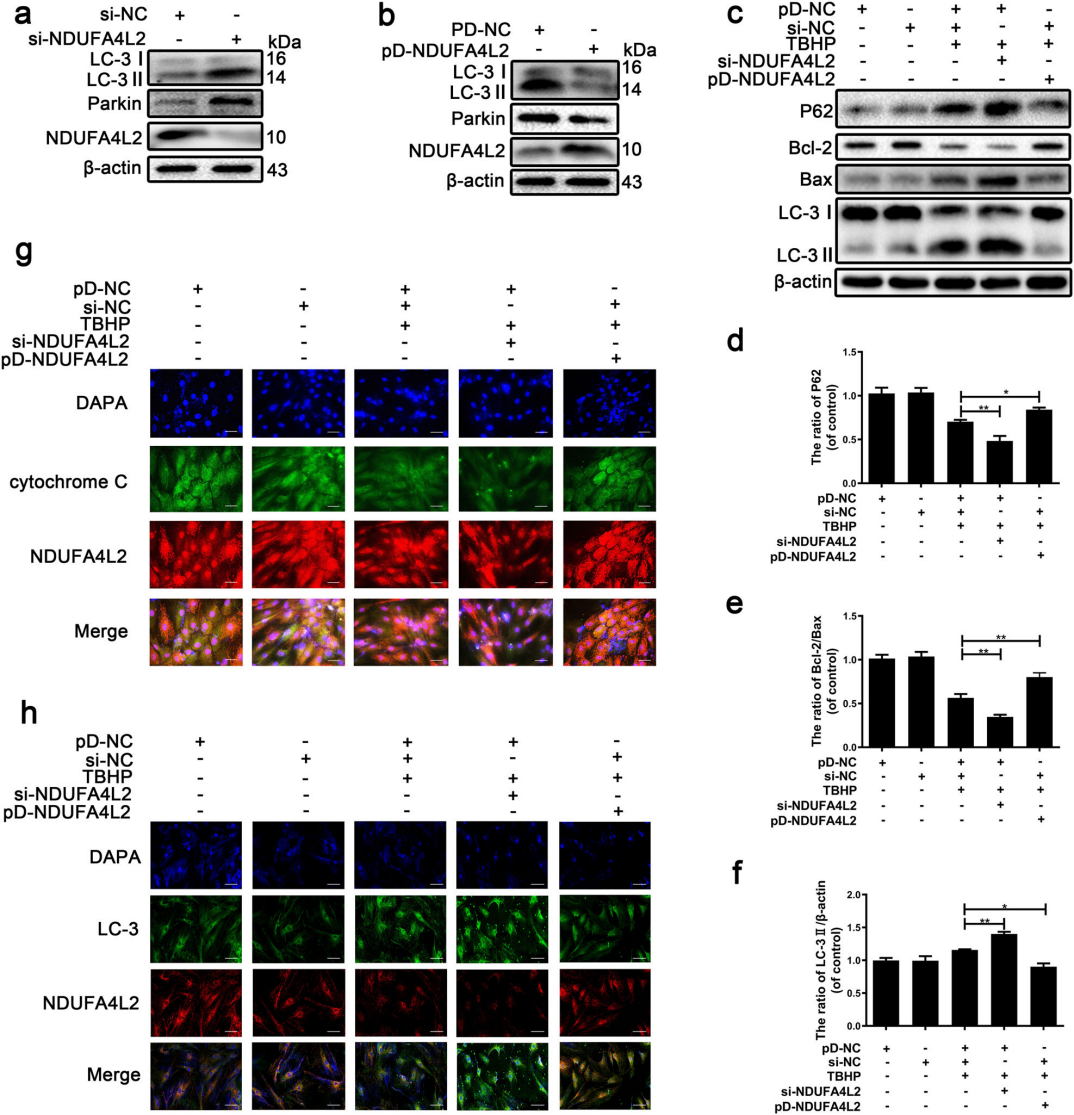
Overexpression of NDUFA4L2 rescues NP cells from apoptosis induced by oxidative stress. Primary nucleus pulposus cells were transfected with si-NDUFA4L2, si-NC, pc-NDUFA4L2 or pc-NC for 48 h and then exposed to TBHP. **a** Western blotting for the protein levels of NDUFA4L2, LC-3II and Parkin was performed to determine the function of si-NDUFA4L2. **b** Western blotting for the protein levels of NDUFA4L2, LC-3II and Parkin was performed to determine the function of pc-NDUFA4L2. **c** Western blotting for the protein levels of NDUFA4L2, LC-3II, Parkin and Bcl-2/Bax was performed to investigate the function of NDUFA4L2 in NP cells exposed to TBHP. **d–f** Quantitative analysis of the protein content of P62, Bcl-2/Bax and LC-3II. **g** Immunofluorescence images showing staining for NDUFA4L2 (red), cytochrome C (green), and DAPI (blue) and merged images of two signals. **h** Immunofluorescence images showing staining for NDUFA4L2 (red), LC-3 (green), and DAPI (blue) and merged images of two signals. The data are presented as the mean ± S.D. ***p < 0.001, **p < 0.01, *p < 0.05 (*n* = 5).

To further confirm our findings, si-NDUFA4L2 and pcDNA-NDUFA4L2 (pc-NDUFA4L2) were designed to repress and promote the protein expression of mitochondrial NDUFA4L2, respectively, because the mitochondrial protein NDUFA4L2 is transported into the cytoplasm. Western blotting revealed that the expression of NDUFA4L2 was knocked down by 80% and overexpressed (Fig. 5a, b). Meanwhile, the protein expression of LC-3II and Parkin was increased in NP cells transfected with si-NDUFA4L2 and decreased in NP cells transfected with pc-NDUFA4L2. These results suggestedthat knockdown and overexpression by si-NDUFA4L2 and pc-NDUFA4L2 were efficient in NP cells.

NDUFA4L2 is a component of the electron transport chain (ETC), and thus, we tried to investigate how decreased NDUFA4L2 impacts the ETC and impairs the mitochondria that induce mitophagy in NP cells. We determined the mitochondrial ROS level by the fluorescent dye H_2_DCFDA in NP cells transfected with si-NDUFA4L2 or pc-NDUFA4L2 and treated with TBHP. The fluorescent dye H_2_DCFDA demonstrated that the level of ROS was markedly higher in NP cells transfected with si-NDUFA4L2 and lower in NP cells transfected with pc-NDUFA4L2 (Fig. S4a and b).

We also measured the phenotype of mitophagy in NP cells to further study whether the absence of NDUFA4L2 causes excessive mitophagy. Figure 5c shows that mitophagy was increased in NP cells transfected with si-NDUFA4L2. However, mitophagy was reduced when NDUFA4L2 was overexpressed in NP cells (Fig. 5c, d, f). Western blotting showed that the overexpression of NDUFA4L2 decreased apoptotic cell death, demonstrating that NDUFA4L2 regulated TBHP-induced cytotoxicity (Fig. 5c, e). Coimmunofluorescence analysis demonstrated that NDUFA4L2 was located in mitochondria and that the overexpression of NDUFA4L2 protected mitochondria against mitophagy (Fig. 5g, h). As shown in the pD-NC + si-NDUFA4L2 groups, Cyt-c was translocated to the cytoplasm during cell apoptosis that Cyt-c and NDUFA4L2 were not colocalized in mitochondria. These results showed that cells in which Cyt-c and NDUFA4L2 were not colocalized in mitochondria were undergoing apoptosis. In conclusion, these results illustrated that the upregulation of NDUFA4L2 protected NP cells against mitophagy and apoptosis induced by oxidative stress by restraining the production of ROS.

### Restraint of excessive mitophagy attenuates the apoptosis of NP cells in the absence of NDUFA4L2

**Fig. 6:**
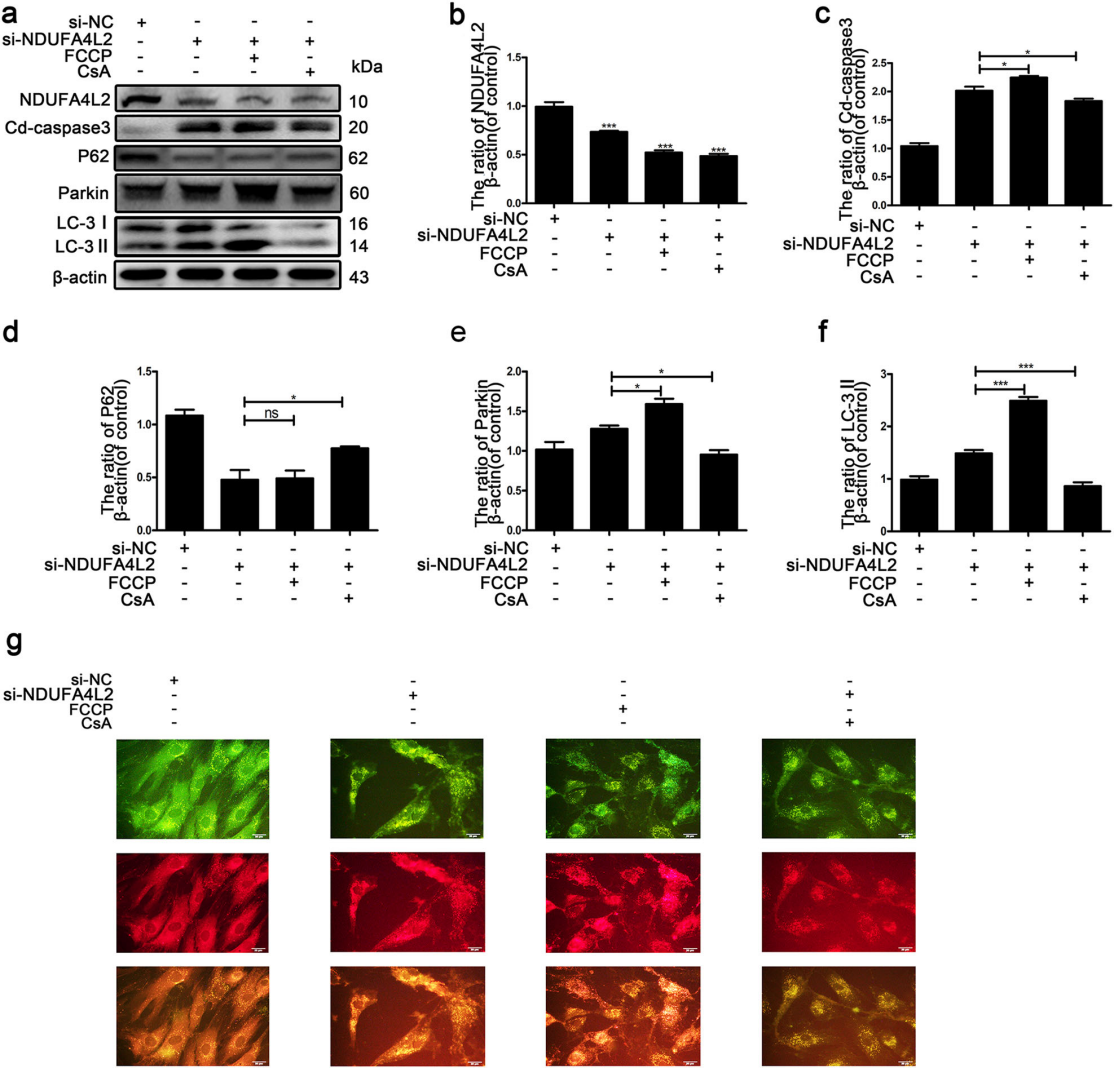
NDUFA4L2 regulates mitophagy induced by oxidative stress. Primary nucleus pulposus cells were transfected with si-NDUFA4L2 or si-NC for 48 h and then exposed to FCCP or CsA. **a** Western blotting for the protein levels of NDUFA4L2, cleaved caspase-3, P62, Parkin and LC-3II. **b**–**f** Quantitative analysis of the protein content of NDUFA4L2, cleaved caspase-3, P62, Parkin and LC-3II. **g** Fluorescence images of NP cells infected with mRFP-GFP-LC-3 adenovirus. sensGFP is sensitive to the pH changes due to the fusion of autophagosomes and lysosomes, whereas mRFP is stable. When autophagy was induced, autophagosomes and lysosomes were fused, sensGFP was quenched and mRFP was increased. The data are presented as the mean ± S.D. ***p < 0.001, **p < 0.01, *p < 0.05 (*n* = 5).

To investigate whether the inhibition of excessive mitophagy attenuates the apoptosis of NP cells upon the silencing of NDUFA4L2, NP cells transfected with si-NDUFA4L2 were cultured with FCCP or CsA for 1 h. Figure 6g shows that mitophagy was repressed by CsA and promoted by FCCP. Western blotting also revealed that the protein expression of LC-3II and Parkin was increased and the expression of P62 was decreased in NP cells treated with CsA (Fig. 6a, d–f). The apoptotic marker cleaved caspase-3 was decreased in NP cells treated with CsA (Fig. 6c). These results indicated that inhibition of excessive mitophagy attenuated the apoptosis of NP cells in the absence of NDUFA4L2.

### Upregulation of NDUFA4L2 alleviates IVDD in rats in vivo

**Fig. 7:**
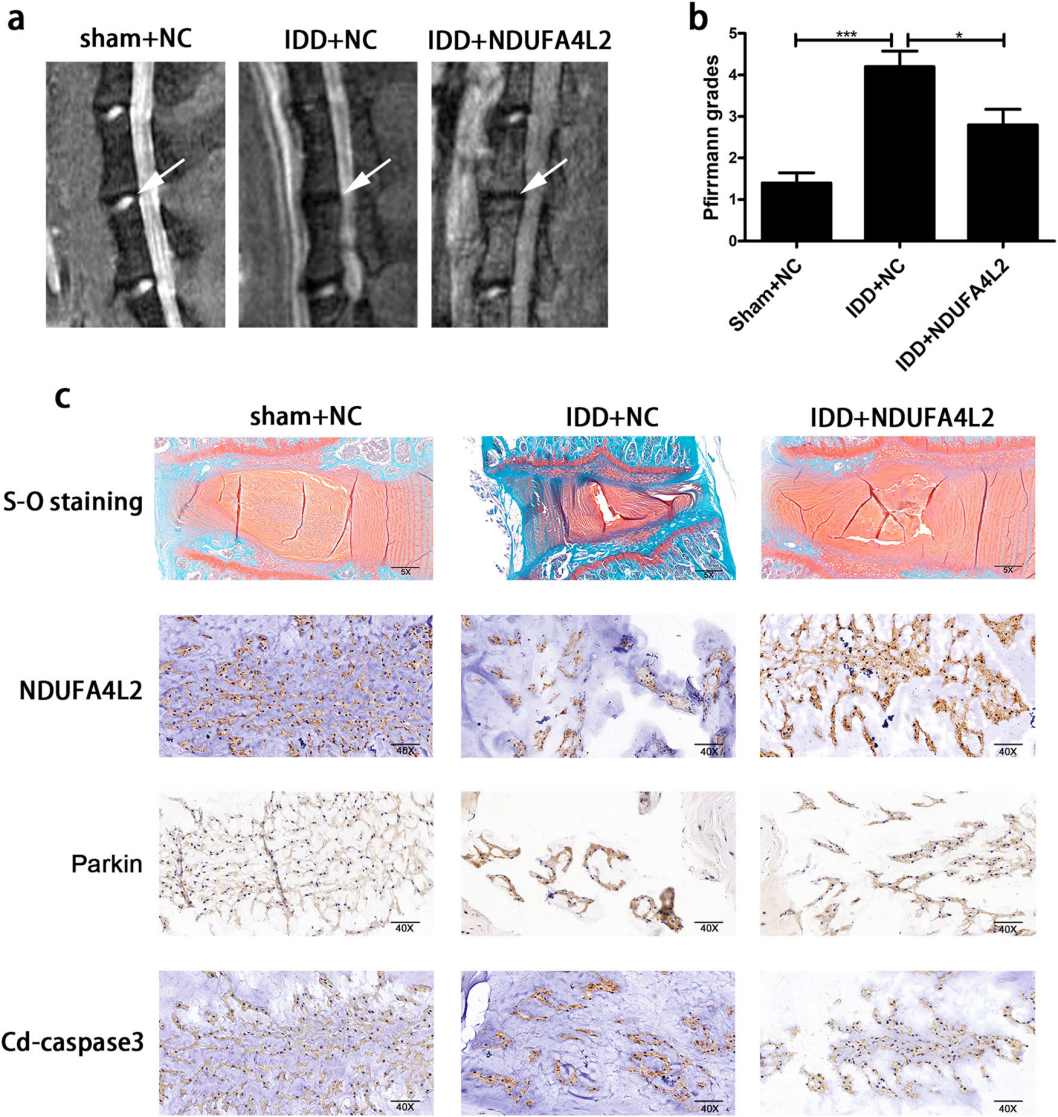
Overexpression of NDUFA4L2 attenuates IVDD in vivo. **a** T2-weighted MRI of IVDD models from each group at 6 months. **b** Pfirrmann MRI scores for T2-weighted MRIs of IVDD models from each group at 6 months. The data are presented as the mean ± S.D. ***p < 0.001, **p < 0.01, *p < 0.05 (*n* = 5). **c** Representative S-O staining of disc samples. Immunohistochemical staining of NDUFA4L2, Parkin and cleaved caspase-3 expression in the disc samples.

We detected the expression of NDUFA4L2, Parkin, and cleaved caspase-3 in a dynamic model established by the injection of adeno-NC (adenovirus-NC) or adeno-NDUFA4L2 (adenovirus-NDUFA4L2) by immunohistochemical staining. We used magnetic resonance imaging (MRI) and Pfirrmann MRI grade scores to assess the different levels of IVDD in rats 6 months after surgery. MRI showed that intervertebral disc degeneration was alleviated in IVDD models injected with adeno-NDUFA4L2 (Fig. 7a, b). Immunohistochemical staining showed that the protein expression of NDUFA4L2 was increased in IVDD models injected with adeno-NDUFA4L2 (Fig. 7c). The protein levels of Parkin and cleaved caspase-3 were markedly higher in dynamic models injected with adeno-NC than in sham models injected with adeno-NC. However, these parameters were obviously lower in IVDD models injected with adeno-NDUFA4L2 than in IVDD models (Fig. 7c). The results confirmed our in vitro findings that the upregulation of NDUFA4L2 facilitated the survival of NP cells and alleviated the levels of IVDD.

### The HIF-1α/NDUFA4L2 pathway is involved in human IVDD

**Fig. 8:**
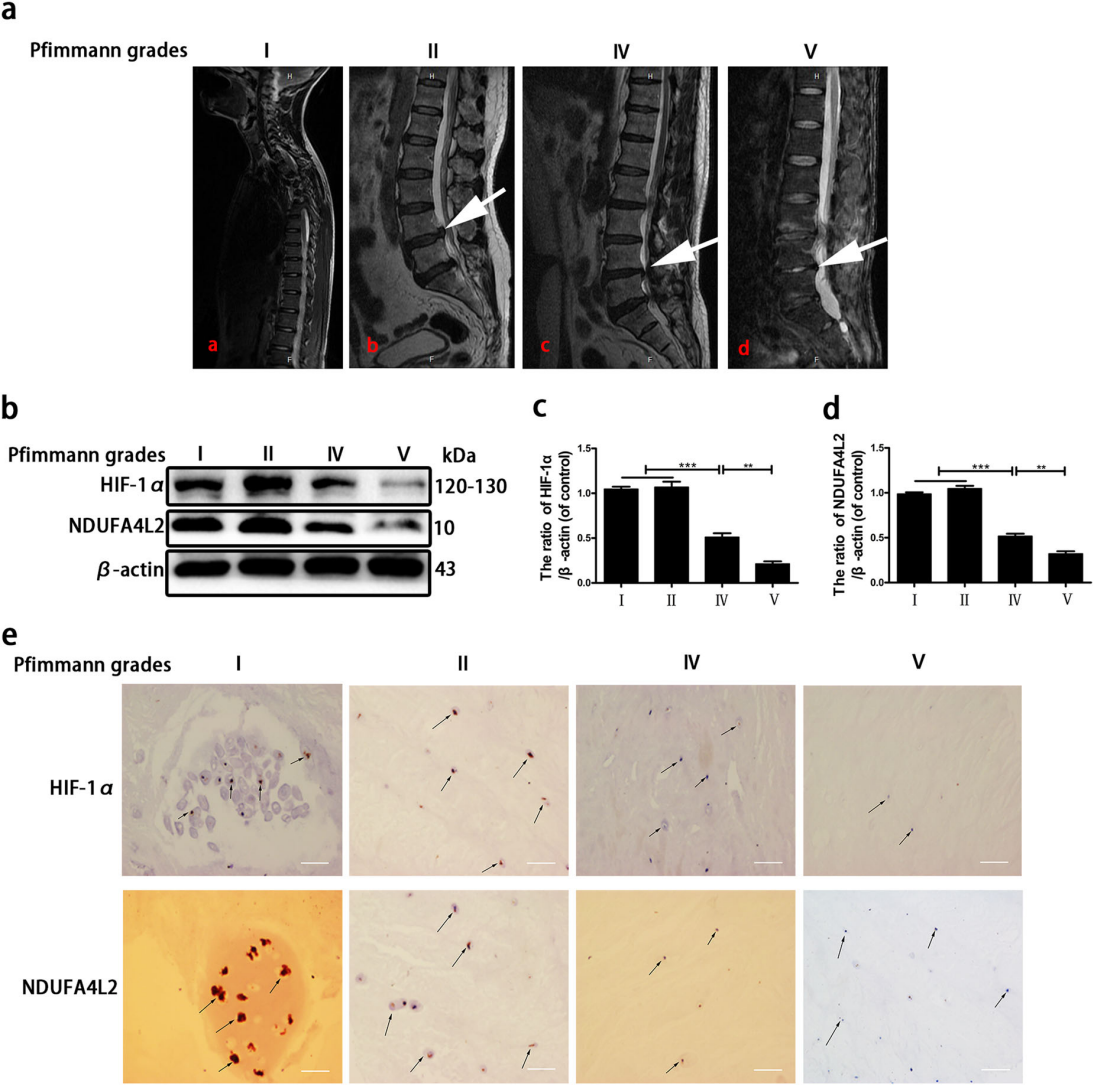
HIF-1α/NDUFA4L2 pathway is decreased as the degree of disc degeneration increases. **a** Pfirrmann grade I patient, male, 15 years old, congenital scoliosis. **b** Pfirrmann grade II patient, male, 61 years old, lumbar disc herniation. **c** Pfirrmann grade IV, 65 years old, lumbar disc herniation. **d** Pfirrmann grade V patient, 63 years old, lumbar disc herniation. **b** Western blotting for the expression of HIF-1α and NDUFA4L2 in different degrees of disc herniation (Pfirrmann grades I, II, IV and V). **c** A bar diagram of HIF-1α expression in each human NP tissue group (Pfirrmann grades I, II, IV and V). **d** A bar diagram of NDUFA4L2 expression in each human NP tissue group (Pfirrmann grades I, II, IV and V). **e** Immunohistochemical examination of the expression of HIF-1α and NDUFA4L2. The data are presented as the mean ± S.D. ***p < 0.001, **p < 0.01, *p < 0.05 (*n* ≥ 3).

The above results indicated that the HIF-1α/NDUFA4L2 pathway regulates the apoptosis of NP cells. To further verify the role of the HIF-1α/NDUFA4L2 pathway in human IVDD, we detected the expression of HIF-1α and NDUFA4L2 in discs with different degrees of degeneration by Western blotting and immunohistochemistry. Three grade I, 5 grade II, 5 grade IV and 5 grade V samples were collected and classified according to the Pfirrmann grade system^30^ (Fig. 8a). Western blotting showed that the expression of HIF-1α and NDUFA4L2 was decreased as the degree of disc degeneration increased (Fig. 8b–d). Immunohistochemistry confirmed the results of Western blotting (Fig. 8e). These results confirmed that the HIF-1α/NDUFA4L2 pathway plays a significant role in human IVDD.

## Discussion

A better understanding of the pathogenesis of IVDD can provide new effective therapies for low back pain (LBP). The apoptosis of NP cells is a key factor in the pathogenesis of apoptosis. Nevertheless, the role of apoptosis induced by strong oxidative stress in IVDD remains unclear.

As an important type of cell death, apoptosis is thought to play an important role in the process of degeneration^7^. Intervertebral discs lack sufficient cells to produce ECM due to the apoptosis of NP cells and AF cells. Many factors affect the apoptosis of the intervertebral disc, especially strong oxidative stress. High levels of ROS are observed in severe disc degeneration^6^. To investigate the mechanism of apoptosis induced by strong oxidative stress, NP cells were exposed to 400 μM TBHP for 6 h. We confirmed that the apoptosis of NP cells was increased by treatment with TBHP. These results proved that strong oxidative stress caused NP cell apoptotic death and induced IVDD.

Autophagy is also induced by oxidative stress, which removes damaged organelles. An appropriate level of autophagy protects cells against pathophysiological injury, while excessive autophagy induces cell apoptotic death^31^. Our previous studies also proved that appropriate autophagy protects disc cells against apoptosis, while excessive autophagy induces apoptosis^23,31^. Autophagy includes macroautophagy, microautophagy and mitochondrial autophagy. Mitochondrial autophagy, known as mitophagy, refers to the process by which cells selectively remove excess or damage mitochondria through autophagy^32^. However, excessive mitophagy causes the apoptosis of cells^33,34^. To further investigate whether mitophagy removed mitochondria, resulting in apoptosis, NP cells were pretreated with FCCP or CsA. CsA markedly decreased LC-3□, Parkin, and cleaved caspase-3 expression and increased the ratio of Bcl-2/Bax, which revealed that the downregulation of mitophagy alleviated NP cell apoptosis.

The mechanism of excessive mitophagy in NP cells remains to be determined. We speculate that HIF-1α is a mediator that regulates mitophagy induced by oxidative stress, based on our previous study^35^. HIF-1α is overexpressed in a hypoxic environment and regulates cell metabolism to adapt to a low oxygen environment^36^. Nevertheless, recent studies have reported that HIF-1α not only plays a vital role in hypoxia but also has a significant influence on cells residing in normoxic environments. HIF-1α is also highly expressed in normoxic environments^37,38^. Importantly, our results demonstrated that the protein expression of HIF-1α was decreased in NP cells cultured with TBHP. To further investigate the role of HIF-1α, NP cells were treated with digoxin or si-HIF-1α⊡ Treatment with digoxin and the silencing of HIF-1α promoted the level of mitophagy, and the apoptosis of NP cells was also increased. However, the inhibition of mitophagy by CsA prevented the effect of digoxin. Moreover, the function of mitochondria was determined by JC-1 staining, and the mitochondrial membrane potential was decreased, which indicated mitochondrial damage. These experiments demonstrated that mitophagy and apoptosis were caused by oxidative stress through the repression of the expression of HIF-1α in NP cells.

Mitochondrial NDUFA4L2, a downstream target of HIF-1α, is a component of ECL that controls oxidative phosphorylation. Mitochondrial ROS are mainly produced by the oxidative respiratory chain and cause damage to mitochondria. Impaired mitochondria are phagocytosed and removed by autophagosomes. Our results confirmed that HIF-1α regulated mitophagy and apoptosis caused by oxidative stress through NDUFA4L2. The protein expression of NDUFA4L2 was lower in human degenerative intervertebral discs than in normal intervertebral discs. The protein expression of NDUFA4L2 was also decreased in NP cells exposed to TBHP. Importantly, the inhibition of HIF-1α repressed the protein expression of NDUFA4L2. The silencing of NDUFA4L2 accelerated the level of mitophagy and apoptosis, while the upregulation of NDUFA4L2 promoted the survival of NP cells cultured with TBHP. The production of mitochondrial ROS was restrained after the overexpression of NDUFA4L2. We found that the inhibition of mitophagy also prevented NP cells from apoptosis induced by silencing NDUFA4L2.

To further confirm the role of NDUFA4L2 in vivo, we established an unbalanced dynamic model. Magnetic resonance images showed that the overexpression of NDUFA4L2 alleviated the degeneration of the intervertebral disc. The expression of Parkin and cleaved caspase-3 showed that mitophagy and apoptosis were decreased. The outcomes indicated that the upregulation of NDUFA4L2 facilitated the survival of NP cells and alleviated the levels of IVDD. The role of mitophagy in IVDD should be confirmed in follow-up experiments in vivo.

The interplay between autophagy and apoptosis is complex and controversial and depends on the types of cells and stresses^39^. An appropriate level of autophagy facilitates the survival of cells, while excessive autophagy promotes apoptotic cell death. A study by Chen et al.^11^ showed that autophagy rescues apoptosis caused by mild oxidative stress. In their research, the concentration of TBHP (100 μM) was much lower than that used in our study (400 μM). In addition, the exposure time of TBHP (4 h) was also markedly shorter than that used in our study (6 h). In another study, Zhang et al.^40^ demonstrated that mitophagy has a protective role in NP cells exposed to TNF-α. Their results showed that the level of mitophagy is increased in human degenerative intervertebral discs. This finding is consistent with our results and viewpoints. However, the promotion of mitophagy did not prevent IVDD, which indicated that mitophagy might protect NP cells at the early stage of disc degeneration. Furthermore, the amount of TBHP added to NP cells was much higher than that of TNF-α. We studied the late stages of IVDD. Hence, our results do not conflict with their studies.

In conclusion, the mechanisms of IVDD remain unknown and require further illumination. Our study demonstrated that the apoptosis of nucleus pulposus cells induced by oxidative stress was regulated by the HIF-1α/NDUFA4L2 pathway, whereas the inhibition of mitophagy by CsA protected nucleus pulposus cells against oxidative stress and apoptosis. Along with further research, modulating mitochondrial NDUFA4L2 and mitophagy in nucleus pulposus cells under oxidative stress might be a potential therapeutic strategy for IVDD.

## Supplementary information

Supplementary material
