# Supplementary material for: Mitochondrial NDUFA4L2 attenuates the apoptosis of nucleus pulposus cells induced by oxidative stress via the inhibition of mitophagy
Source: Exp Mol Med. 2019 Nov 18;51(11):140. doi: 10.1038/s12276-019-0331-2 (PMC6861227; doi:10.1038/s12276-019-0331-2)
Supplement: Supplementary file 1 — Supplementary material [file 12276_2019_331_MOESM1_ESM.docx]

**
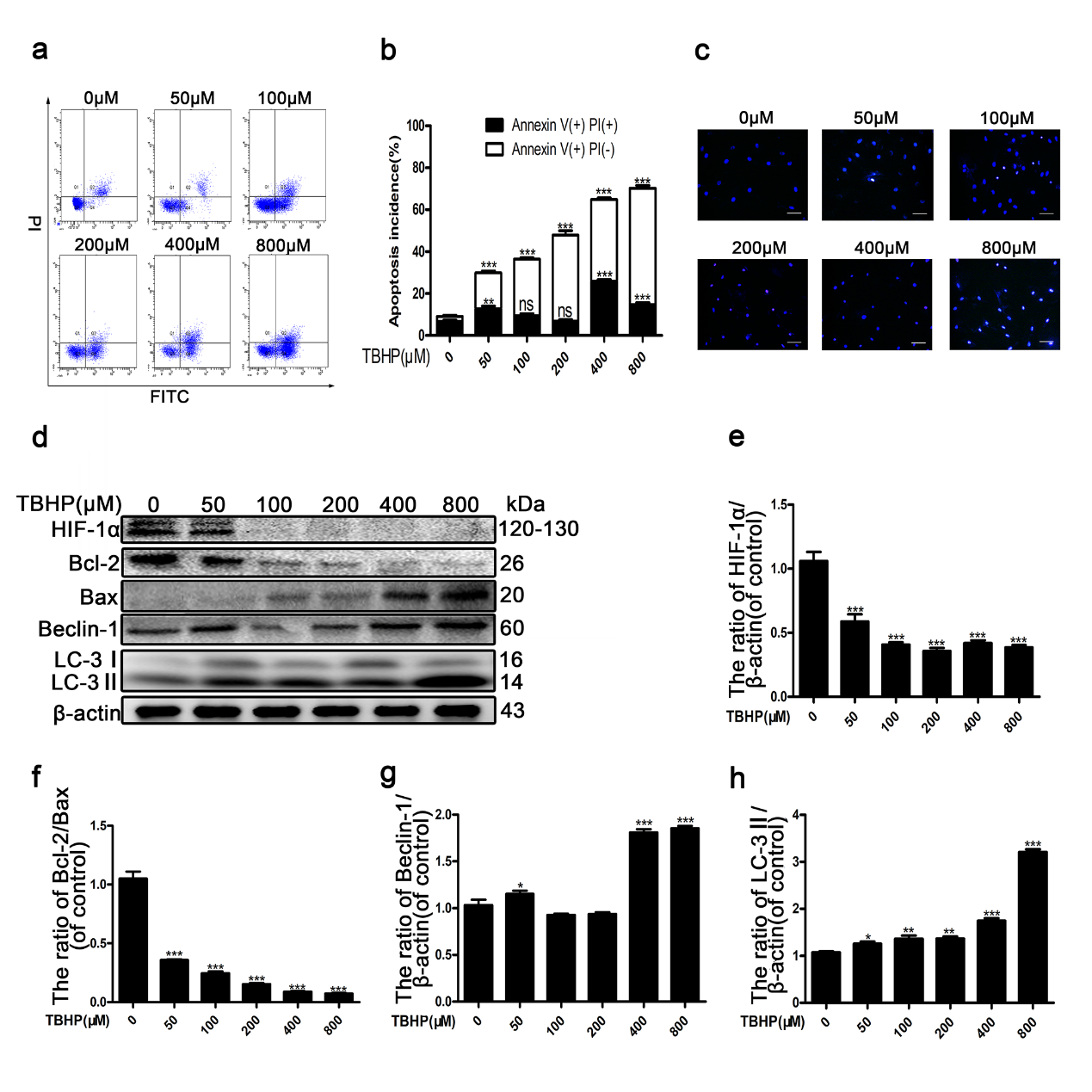
**

**Figure S1** Primary nucleus pulposus cells were cultured in different concentrations of TBHP for 6h. (a) Flow cytometry to detect the apoptosis of nucleus pulposus cells. (b) Quantitative analysis of flow cytometry. (c) Hoechst 33258 staining detect the apoptosis of nucleus cells cultured in different concentrations of TBHP for 6h. Nuclear condensation was observed in the apoptotic cell. (d) Western blotting for the protein level of HIF-1α, Bcl-2/Bax, Beclin-1 and LC-3Ⅱ. (d-h) Quantitative analysis for the protein content of HIF-1α, Bcl-2/Bax, Beclin-1 and LC-3Ⅱ. The data were represented by averages±S.D. ***p < 0.001, **p < 0.01, *p < 0.05 (n=5).

**
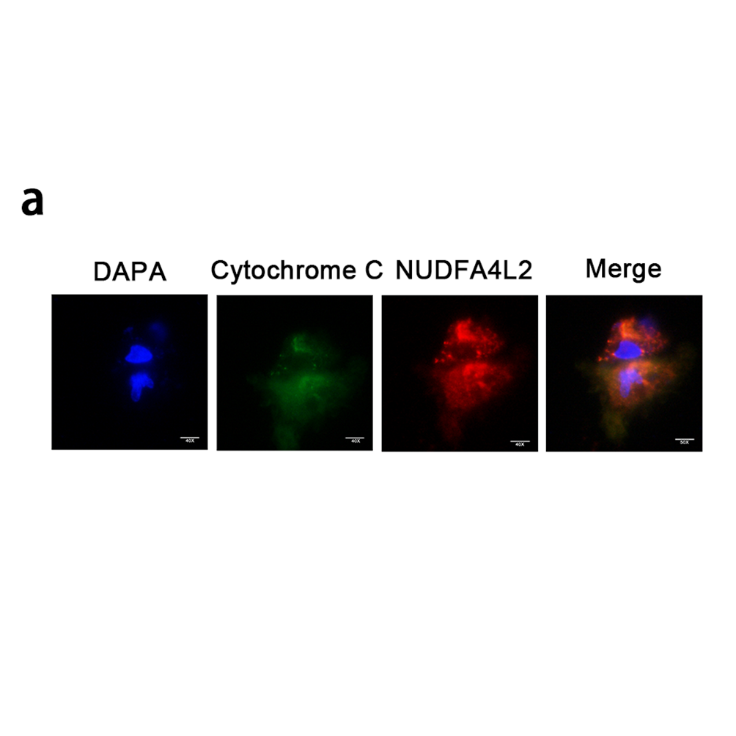
**

**Figure S2** (a) Immunofluorescence images showed staining for NDUFA4L2 (red), cytochrome C (green), DAPA (blue) and Merge images of the two signals (n=5).


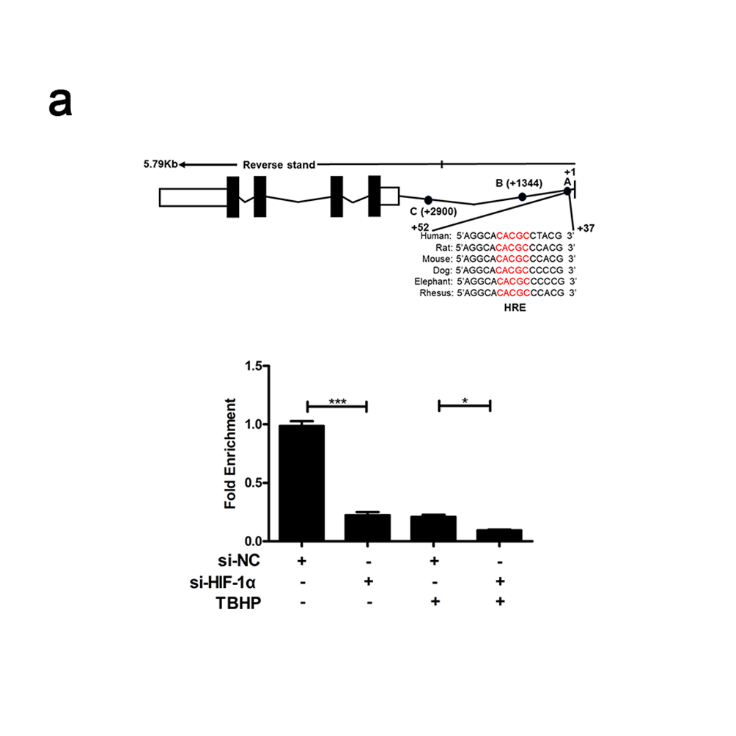


**Figure S3** (a) Schematic representation of NDUFA4L2 gene and the nucleotide sequences matching the consensus hypoxia response element (HRE) from six mammalian genes, indicating the regions A analyzed in the CHIP assay. (b) CHIP assay of HIF-1α binding to NDUFA4L2 gene in rat NP cells. (n=5).


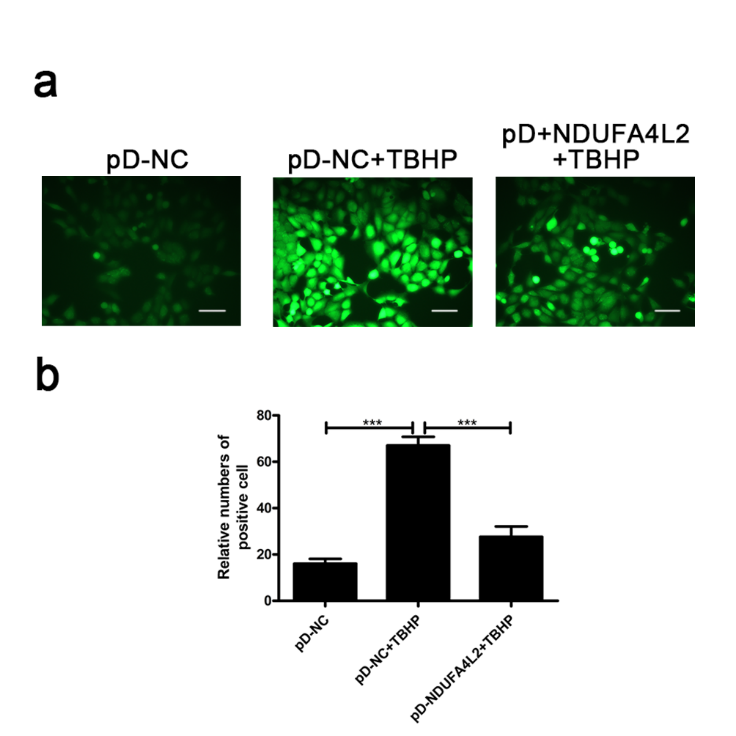


**Figure S4** (a) Mitochondrial production of ROS was detected by Reactive Oxygen Detection Kit. Green dot represented the ROS was increased in NP cells (n=5).
